# Supplementary material for: Belonging to Three Worlds: Somali Adolescent–Parent Relationships in the United States and Implications for Tobacco Prevention
Source: Int J Environ Res Public Health. 2022 Mar 19;19(6):3653. doi: 10.3390/ijerph19063653 (PMC8953780; doi:10.3390/ijerph19063653)
Supplement: Supplementary file 1 [file ijerph-19-03653-s001.zip › ijerph-1590260-supplementary.pdf]

Date: ______________________ DVR# Folder Msg #
Research Staff Initials: _________ Primary Recording _____ _____ _____
KI-ID#: ______________________ Back-up Recording _____ _____ _____

Supplementary File S1: Key Informant Interview Guide

INTRODUCTORY QUESTIONS:

1. Briefly describe your experiences working with Somali youth here in the Twin Cities at [organization/agency].

2. How would you rank tobacco use relative to other major threats to the health of Somali youth growing up here?

   a. Tell me more about why you rank tobacco use there?

3. Describe some key cultural values that the Somali communities hold for their adolescents.

   a. Can you give an example of how these values influence Somali youth decisions regarding tobacco use?

DETERMINANTS OF TOBACCO USE:

4. What would you say are the most significant influences on Somali youth decisions about using tobacco?

   Probes:

   a. In your opinion, which of these factors are the most important?
   b. How, if at all, have you seen Somali youth beliefs about the potential benefits or risks of tobacco products influence their decisions to use tobacco? Can you provide a specific example of a time when you observed this directly?
   c. How, if at all, does **being a boy or girl** influence Somali youth tobacco use?
   d. Are some forms of tobacco more acceptable than others (such as shisha or hookah) in the Somali communities? How do Somali communities view *youth* tobacco use? Share with me an example of how this played out.
   e. Share an example of how **religious faith** influences Somali youth tobacco use?

COMMUNITY ASSETS AND BARRIERS:

5. Where do Somali youth find sources of support within their communities? (e.g., parents, other family, friends, teachers/schools, coaches, religious leaders/institutions, others?)

   Probes:

a. Which of these supports are most important to addressing youth tobacco use? Why?

6. What are some of the biggest challenges to youth tobacco prevention currently facing Somali communities here?

Probes:

a. What are the major contributors to these challenges?

PARENT ROLES:

7. While parents play key supportive roles for their adolescent children, many parents find that it is challenging to raise teenagers. Tell me about Somali parents' experiences raising teenagers. What is it like for them?

Probes:

a. What are some of the main challenges that Somali parents face raising teenagers?
b. What additional parenting resources or supports might benefit Somali parents of teens?

POTENTIAL RESPONSES/SOLUTIONS:

8. We spoke earlier about key influences in the lives of Somali young people including [parents, peers, religion, culture, etc.]. Reflecting on these, what would your dream program to prevent tobacco use among Somali adolescents look like? Assume that funding is not a concern.

Probes:

a. Who would the program include (e.g., parents, other family members, youth)? How might the program involve both fathers and mothers?
b. What topics would be most important to cover (e.g., for parents topics on parenting Somali teens in the U.S., etc.)?
c. Which values and traditions should be addressed?
d. What aspects of religious faith would resonate best with participants (e.g., messages delivered by religious leaders, religious framing, etc.)?
e. What would the ideal format for the program be (e.g., one-on-one, group, in-person, online)?
f. Where would you envision a program like this taking place? Why? What would make the location more welcoming?
g. Are there ideal characteristics of the facilitator (e.g., bilingual, known in the community, a health professional)?

9. What additional information do you think it would be important to know about Somali youth tobacco use when designing prevention initiatives?
